# Salvage brachytherapy for seminal vesicle recurrence after initial brachytherapy for prostate cancer: a case report

**DOI:** 10.1186/1756-0500-7-760

**Published:** 2014-10-26

**Authors:** Shunta Hori, Nobumichi Tanaka, Isao Asakawa, Yosuke Morizawa, Akihide Hirayama, Masatoshi Hasegawa, Noboru Konishi, Kiyohide Fujimoto

**Affiliations:** Department of Urology, Nara Medical University, 840 Shijo-cho, Kashihara, Nara, 634-8522 Japan; Department of Radiation Oncology, Nara Medical University, Nara, Japan; Department of Urology, Nara Hospital of Kinki University, Nara, Japan; Department of Pathology, Nara Medical University, Nara, Japan

**Keywords:** Salvage brachytherapy, Seminal vesicle recurrence, Prostate cancer

## Abstract

**Background:**

To report the efficacy and safety of salvage brachytherapy for seminal vesicle recurrence after initial brachytherapy in a patient with prostate cancer. As far as we know, this is a first report of salvage brachytherapy for seminal vesicle recurrence in Japan.

**Case presentation:**

A 70-year-old Japanese man with low-risk prostate cancer received low-dose-rate brachytherapy. Forty-two months after the seed implantation, he showed biochemical recurrence based on the nadir + 2 ng/mL definition. The prostate specific antigen (PSA) level was 5.11 ng/mL at 58 months after seed implantation. A saturation biopsy of the prostate showed no recurrence. Systemic screening also showed no distant metastases. However, T2-weighted magnetic resonance imaging (MRI) demonstrated a low intensity area at the base of the right seminal vesicle, which was strongly suggestive of recurrence. Sixty months after the initial therapy, a seminal vesicle biopsy confirmed recurrence with a Gleason score of 4 + 3 before salvage brachytherapy was performed. The prescribed dose was 145 Gy, the same as the dose of the initial therapy. One month later, the PSA level had rapidly declined to 0.898 ng/mL without androgen deprivation therapy. Ten months after the salvage brachytherapy, the PSA level reached 0.078 ng/mL. No adverse events were seen during the follow-up period.

**Conclusions:**

We experienced a patient who was successfully treated with salvage brachytherapy for seminal vesicle recurrence. Salvage brachytherapy is one of the promising therapeutic options for recurrence after initial brachytherapy.

## Background

Several salvage therapeutic options including radical prostatectomy, radiation therapy and androgen deprivation therapy can be considered for patients who show seminal vesicle recurrence after prostate brachytherapy. We experienced a patient who underwent salvage brachytherapy. This is the first report of a case of salvage brachytherapy for seminal vesicle recurrence after prostate brachytherapy in Japan, as far as we know.

## Case presentation

**Figure 1:**
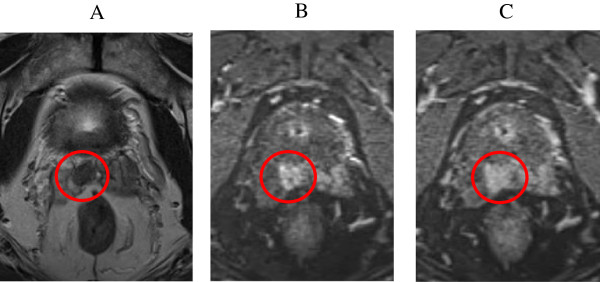
**MRI images of seminal vesicle recurrence. (A)** T2-weighted image demonstrated a low-intensity area in the bottom of seminal vesicle. **(B, C)** Dynamic MRI also demonstrated a recurrence of seminal vesicle because of an intensive enhancement in early phase and a washed-out in late phase.

**Figure 2:**
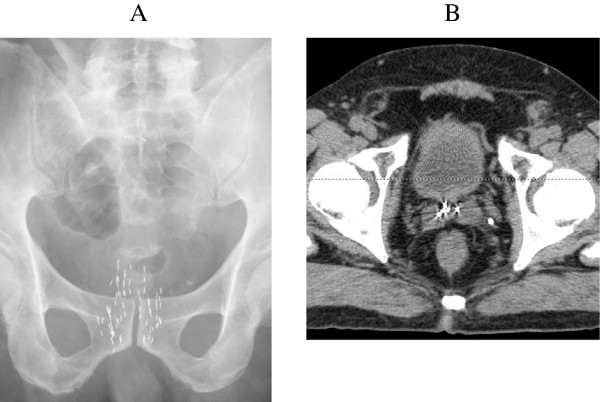
**Pelvic X-ray and CT scan images after salvage brachytherapy. (A)** Pelvic X-ray and **(B)** CT scan demonstrated the source at the bottom of seminal vesicle.

A 70-year-old Japanese man underwent a medical checkup in March 2005, which showed an abnormal prostate specific antigen (PSA) value (4.8 ng/mL), and he consulted a urologist. He underwent needle biopsy of the prostate. The histopathological findings indicated an adenocarcinoma with a Gleason score of 3 + 3. The number of positive cores was 4 out of 8 cores (right lobe: 1/4, left lobe: 3/4). The digital rectal examination showed no abnormal findings. The clinical stage was T1cN0M0. In 2006, he underwent a low-dose-rate brachytherapy (LDR-brachytherapy) using a preplanning method and modified peripheral loading at our hospital. The prescribed dose was 145 Gy. Regarding dosimetric parameters using CT images 1 month after seed implantation, the minimal dose (Gy) received by 90% of the prostate gland (D90) was 183 Gy, the percentage prostate volume that received 100% of the prescribed minimal peripheral dose (V100) was 98.5%, and the rectal volume (mL) that received 100% of the prescribed dose (R100) was 0.02 mL. One year after seed implantation, the PSA level reached the nadir at 0.38 ng/mL. The PSA level then increased gradually. Forty-two months later, PSA recurrence was confirmed based on the Phoenix definition (nadir + 2 ng/mL). The patient was then observed without any salvage therapy because the dosimetric quality of the initial therapy was excellent and the possibility of PSA bounce was also considered. Fifty-eight months after seed implantation, the PSA level reached 5.11 ng/mL. A prostate saturation biopsy demonstrated no obvious recurrence inside the prostate. Systemic screening by lung and abdominal CT and bone scan did not detect distant metastases. On the other hand, T2-weighted and dynamic MRI images demonstrated a low intensity area at the base of the right seminal vesicle, which was very suspicious of recurrence (Figure 1A,B,C). We proposed salvage prostatectomy, salvage brachytherapy or androgen deprivation therapy to the patient. Sixty months after the initial therapy, he underwent salvage brachytherapy after providing informed consent. The prescribed dose of the salvage therapy was 145 Gy as same as the initial therapy. We used a radioactive source of 0.33 mC1 (13.1 MBq) for salvage brachytherapy (as same as initial brachytherapy). Prior to salvage brachytherapy, seminal vesicle biopsy was performed and the histological findings showed an adenocarcinoma with a Gleason score of 4 + 3 at the base of the right seminal vesicle. The base of right seminal vesicle was set as the planning target volume (PTV) with 1 cm margin. Seven needles were inserted into the PTV, and 2 or 3 sources were implanted through each needle. Finally 20 sources were implanted by the real-time planning method (Figure 2). Regarding dosimetric parameters using CT images 1 month after salvage brachytherapy, seminal vesicle D90 was 280 Gy, seminal vesicle V100 was 100%, prostate D90 was 206.4 Gy, prostate V100 was 99.2%, the R100 was 0.26 mL, and the minimal dose (Gy) received by 30% of the urethra (UD30) was 285.7 Gy. The treatment time was 69 minutes. Since the PSA level rapidly decreased to 0.898 ng/mL by 1 month after seed implantation, he received no adjuvant therapy. Ten months after seed implantation, the PSA level reached 0.078 ng/mL. No adverse events were seen during the follow-up period.

## Discussion

When patients show PSA recurrence after LDR-brachytherapy, there are several options for salvage treatments such as androgen deprivation therapy, radical prostatectomy, radiation therapy, etc. A 70-year-old Japanese man has a life-expectancy of 15.1 years if no severe co-morbidities are present. We discussed the merits and demerits of each modality of salvage therapy with the patient. He finally chose salvage brachytherapy of the seminal vesicle. There have been several previous reports of salvage brachytherapy (Table 1). The oncological outcomes showed a biochemical recurrence-free rate of around 70%. Some patients have showed genitourinary and gastrointestinal adverse events of grade 3 or greater, but the incidence rate was acceptable. Some reports involved prescription of a full dose (145 Gy), while the prescribed dose was reduced according to other reports [1–7]. There is not a standard prescribed dose and the pros and cons of adjuvant hormonal therapy are not established. As far as we know, there are two reports of salvage brachytherapy for seminal vesicle recurrence. Koutrouveil *et al*. reported 31 patients who showed recurrence after initial brachytherapy. Eleven of the 31 patients showed recurrence in the seminal vesicle, and 9 of these 11 patients showed no recurrence after salvage brachytherapy. They also showed that salvage brachytherapy was as useful as other salvage therapies because a high biochemical control rate (87%) was achieved [1]. Burri *et al*. also reported the results of salvage brachytherapy in 37 patients. They concluded that a PSA level of less than 6 was a prognostic factor for salvage brachytherapy in multivariate analysis [2]. However, large-scale studies on salvage brachytherapy have not been published. Therefore, patient selection, optimal prescribed dose, and use of adjuvant androgen deprivation therapy are still controversial. The excessive dose area of the seminal vesicle due to the small target volume may consequently lead to the problem that the urethral dose and the dose to the bottom of the prostate are apt to be high. It is also difficult to insert needles into the seminal vesicle because the distance from the perineum to the seminal vesicle is longer than to the prostate for seed implantation. With regard to the adverse events of salvage brachytherapy, the RTOG 0526 study is presently in progress and the results are awaited. Our patient shows a favorable outcome at present during the early stage after salvage therapy for seminal vesicle recurrence. Longer follow-up data involving more patients are needed to establish the usefulness of salvage brachytherapy, not only for seminal vesicle recurrence, but also for the primary focus.

**Table 1:** **Several previous reports of salvage brachytherapy**

Study year	N	Prior RT	Use of ADT	Median f/u	Outcome	Adverse event	Prescribed dose
Koutrouveils* 2003 [1]	31	seed	Neo 3 mos except 1	30 mos	5 yr-bDFS: 87%	GI: G4 (2/31)	^125^I:100-144 Gy ^103^Pd:100-120 Gy
Wong 2006 [3]	17	EBRT	Neo 3 mos: 12 ad 6 mos: 5	44 mos	4 yr-bDFS: 75%	GU:G4 (1/17)	^125^I:126 Gy in principle
Nguyen 2007 [4]	25	EBRT: 13 seed: 12	none	47 mos	4 yr-bDFS: 70%	GU: G3-4 13% GI: G3-4 30%	^125^I:137 Gy
Lee 2007 [5]	21	EBRT	Concurrent -ad: 12	36 mos	5 yr-bDFS: 38%	G3 or over: none	^125^I:90 Gy
Aaronson 2009 [6]	24	EBRT	Concurrent: 17%	30 mos	3 yr-bDFS: 89.5%	GU: G3 (1/24)	^125^I:144 Gy (108 Gy)
Moman 2010 [7]	31	EBRT: 20 seed: 11	Neo 3 mos: 5	9.2 yrs	5 yr-FFbF: 23%	lateGU: G3 (6/31) lateGI: G3 (2/31)	^125^I:145 Gy
Burri* 2010 [2]	37	EBRT: 32 seed: 5	Neo + ad 6 mos: 31	86 mos	5 yr-FFbF: 64.5%	GI: G4 (1/37)	^125^I:135 Gy

*Report of brachytherapy for recurrence of seminal vesicle.

RT: Radiation Therapy, ADT: Androgen Deprivation Therapy, f/u: follow-up, EBRT : External Beam Radiation Therapy, Seed: Seed Implant (Brachytherapy), Neo: Neoadjuvant Therapy, Ad: Adjuvant Therapy, bDFS: biochemical Disease Free Survival, FFbF: Freedom From biochemical Failure, GI: Gastrointestinal adverse effect, GU: Genitourinary adverse effect, G: Grade.

## Conclusions

We reported a case of salvage brachytherapy for seminal vesicle recurrence with a favorable outcome. It is expected that salvage brachytherapy will play a significant role as a salvage therapeutic modality after radiation therapy including brachytherapy.

## Consent

Written informed consent was obtained from the patient to publish this case report and any accompanying images. A copy of the written informed consent is available for review by the Editor-in-Chief of this journal.

## Abbreviations

LDR-brachytherapy: Low-dose-rate brachytherapy
PSA: Prostate specific antigen
D90: Minimal dose (Gy) received by 90% of the prostate gland
V100: Percentage prostate volume that received 100% of the prescribed minimal peripheral dose
R100: Rectal volume (mL) that received 100% of the prescribed dose
UD30: The minimal dose (Gy) received by 30% of the urethra
PTV: Planning target volume.
